# Anti-sense DNA d(GGCCCC)_n_ expansions in C9ORF72 form i-motifs and protonated hairpins

**DOI:** 10.1038/srep17944

**Published:** 2015-12-03

**Authors:** Anja Kovanda, Matja Zalar, Primož Šket, Janez Plavec, Boris Rogelj

**Affiliations:** 1 Department of Biotechnology, Jožef Stefan Institute, Jamova 39, SI-1000 Ljubljana, Slovenia; 2 Slovenian NMR Centre, National Institute of Chemistry, Hajdrihova 19, SI-1000 Ljubljana, Slovenia; 3 EN-FIST Center of Excellence, Trg OF 13, SI-1000 Ljubljana, Slovenia; 4 Faculty of Chemistry and Chemical Technology, University of Ljubljana, Večna pot 113, SI-1000 Ljubljana, Slovenia; 5 Biomedical Research Institute BRIS, Puhova 10, SI-1000 Ljubljana, Slovenia

## Abstract

The G_4_C_2_ hexanucleotide repeat expansion mutation (HREM) in *C9ORF72*, represents the most common mutation associated with amyotrophic lateral sclerosis (ALS) and frontotemporal lobar degeneration (FTLD). Three main disease mechanisms have been proposed to date: C9ORF72 haploinsufficiency, RNA toxicity, and accumulation of dipeptide repeat proteins. Pure GC content of the HREM potentially enables the formation of various non-B DNA structures such as G-quadruplexes and i-motifs. These structures are proposed to act as promoters and regulatory elements affecting replication, transcription and translation of the surrounding region. G-quadruplexes have already been shown on the G-rich sense DNA and RNA strands (G_4_C_2_)_n_, the structure of the anti-sense (G_2_C_4_)_n_ strand remains unresolved. Similar C-rich sequences may, under acidic conditions, form i-motifs consisting of two parallel duplexes in a head to tail orientation held together by hemi-protonated C^+^-C pairs. We show that d(G_2_C_4_)_n_ repeats do form i-motif and protonated hairpins even under near-physiological conditions. Rather than forming a DNA duplex, i-motifs persist even in the presence of the sense strand. This preferential formation of G-quadruplex and i-motif/hairpin structures over duplex DNA, may explain HREM replicational and transcriptional instability. Furthermore, i-motifs/hairpins can represent a novel pharmacological target for *C9ORF72* associated ALS and FTLD.

The G_4_C_2_ hexanucleotide repeat expansion mutation (HREM), located in the first intron or promoter region of the *C9ORF72* gene on chromosome 9p21 represents the most common mutation associated with amyotrophic lateral sclerosis (ALS) and frontotemporal lobar degeneration (FTLD) in populations of European origin^1^,^2^,^3^. Individuals without the disorder possess on average 2 repeats with some individuals having up to 19, while patients with ALS or FTLD may present with up to 5000 repeats^2^,^3^,^4^,^5^,^6^,^7^.

Three main disease mechanisms have been proposed to date: C9ORF72 haploinsufficiency, RNA toxicity, and accumulation of dipeptide repeat proteins (DPR)^8^. C9ORF72 haploinsufficiency can arise from changes in the transcription and RNA processing rates of the mutation carrying allele^2^. On the other hand, the repeat RNA may be toxic as the accumulating RNA foci may sequester important RNA binding proteins^9^ a mechanism that has already been suggested for myotonic dystrophy^10^. Finally, the RNA transcripts can undergo repeat-associated non-ATG (RAN) translation, mediated by stable hairpin structures rather than an ATG codon. The resulting DPRs form toxic aggregates in various cell and animal models^11^,^12^,^13^. The complexity surrounding the C9ORF72 disease mechanisms is increased even further as the HREM region undergoes transcription also in the anti-sense direction giving rise to anti-sense RNA foci and additional DPRs^11^.

The pure GC content of both strands of HREM potentially enables the formation of various non-B DNA structures and the G-rich sense DNA and RNA strands (G_4_C_2_)_n_ have been shown to form G-quadruplex structures^14^,^15^,^16^. The structure of the C-rich (G_2_C_4_)_n_ anti-sense strand has not been defined to date, however other C-rich sequences have been suggested to form i-motifs, in acidic conditions, that consist of two parallel duplexes in a head to tail orientation held together by hemi-protonated C^+^-C pairs^17^,^18^. Like G-quadruplexes, i-motifs can act as promoters and regulatory elements affecting replication, transcription and translation of the surrounding region^19^,^20^,^21^.

Here we show that d(G_2_C_4_)_n_ repeats can form i-motif as well as protonated hairpin structures even under conditions approaching physiological relevance and in the presence of the complementary strand. This property may contribute to replicative instability of HREM as well as affect the local transcription. Finally, i-motifs/hairpins may prove a novel drug target for *C9ORF72* associated ALS and FTLD, as similar structures have already been shown to be susceptible to targeting with small molecules^22^.

## Results

### CD spectra indicate formation of i-motifs and hairpins

**Figure 1:**
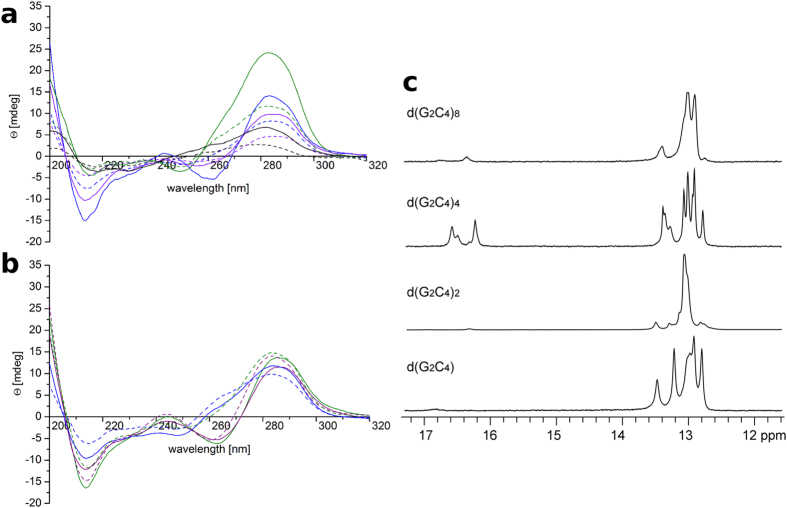
CD and NMR spectra indicate the presence of protonated structures. (**a**) CD spectra of d(G_2_C_4_) (black), d(G_2_C_4_)_2_ (green), d(G_2_C_4_)_4_ (blue), and d(G_2_C_4_)_8_ (purple) immediately after dilution in H_2_O (dashed line) and after incubation (full line). Positive and negative peaks, at 285 and 265 nm, respectively, are associated with the formation of i-motifs. (**b**) Comparison of CD spectra of d(G_2_C_4_)_4_ in water (dashed lines) and under molecular crowding conditions in 40% w/v PEG8000 (full lines) at pH 6.5 (purple), 7.0 (green), 7.2 (blue). Peaks characteristic for i-motifs persist up to pH 7.0 in 40% w/v PEG8000. All spectra were acquired at 37 °C. (**c**) Imino region of ^1^H NMR spectra of d(G_2_C_4_), d(G_2_C_4_)_2_, d(G_2_C_4_)_4,_ and d(G_2_C_4_)_8_ after incubation in H_2_O at pH 6.0 and 5 °C. Signals between δ 12.5 and 13.5 ppm are indicative of Watson-Crick hydrogen bonding, while signals between δ 16.0 and 17.0 ppm correspond to imino protons from hemi-protonated C^+^-C base pairs.

In order to get an indication of the structures formed by the repeats, CD spectra of d(G_2_C_4_), d(G_2_C_4_)_2_, d(G_2_C_4_)_4_, and d(G_2_C_4_)_8_ were measured in deionized H_2_O at 37 °C (Fig. 1a). In case of d(G_2_C_4_)_4_ and d(G_2_C_4_)_8_ a positive peak at 285 nm and a negative peak at 265 nm, characteristic of i-motifs^20^, were clearly visible. CD spectra measured at 5, 25, and 37 °C showed no differences in peak shapes and respective wavelengths (Supplementary Fig. 1). On the other hand the CD spectra of all four investigated oligonucleotides were pH dependent. Spectra of d(G_2_C_4_) did not show any i-motif characteristics at any of the tested pH values (Supplementary Fig. 2), while spectra of d(G_2_C_4_)_2_ and d(G_2_C_4_)_8_ showed a shift toward i-motif characteristic peaks up to pH 5.0 (Supplementary Fig. 3 and 5, respectively) and d(G_2_C_4_)_4_ up to pH 6.5 (Supplementary Fig. 4). However, characteristics of CD spectra as a function of pH could not be entirely explained by a decrease in the population of i-motif structures upon increasing of pH. At pH values higher than 6.5, an additional positive peak at around 260 nm as well as a negative peak at 245 nm were observed while the peak at 285 nm remained. Such observation could be explained by the presence of additional structures, such as hairpins^23^,^24^. The occurrence of additional structures was further substantiated with UV melting (Supplementary Table 1 and Fig. 6, respectively) and native PAGE (Supplementary Fig. 7).

The formation of non-B DNA structures could be affected by the crowded conditions within the nucleus. Because water availability influences the formation of such structures, CD spectra of d(G_2_C_4_)_4_ were then measured under molecular crowding conditions in 40% w/v PEG8000^25^,^26^ at 37 °C and different pH. Under these conditions, CD spectra of d(G_2_C_4_)_4_ exhibited i-motif characteristic peaks up to pH 7.0 (Fig. 1b).

### NMR reveals coexistence of protonated hairpins and i-motifs

Imino region of ^1^H NMR spectra of d(G_2_C_4_), d(G_2_C_4_)_2_, d(G_2_C_4_)_4_, and d(G_2_C_4_)_8_ acquired at pH 6.0 and 5 °C was examined in order to obtain deeper insights into secondary structure formation (Fig. 1c). All four oligonucleotides exhibit signals between δ 12.5 and 13.5 ppm indicative of Watson-Crick hydrogen bonding, which is to be expected given the high GC content and therefore high intra and intermolecular Watson-Crick binding ability of the oligonucleotides. In addition, signals between δ 16.0 and 17.0 ppm were observed, corresponding to imino protons from hemi-protonated C^+^-C base pairs (Fig. 1c). The observed chemical shifts are shifted downfield significantly with respect to previously reported values for protonated cytosines within i-motifs^27^ suggesting formation of other protonated structures.

**Figure 2:**
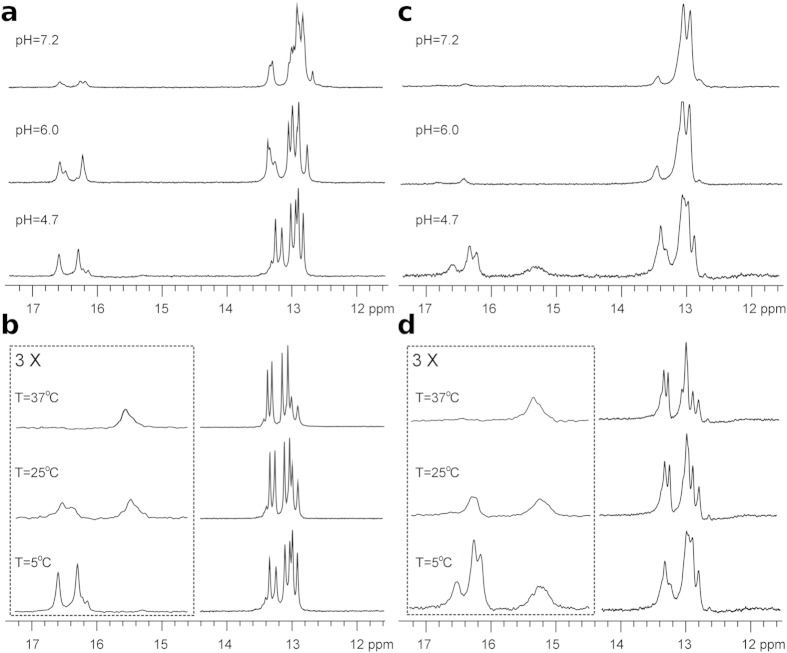
Coexistence of i-motifs and protonated hairpins. (**a**) Imino region of ^1^H NMR spectra of d(G_2_C_4_)_4_ at 5 °C and pH 4.7, 6.0, and 7.2. (**b**) Imino region of ^1^H NMR spectra of d(G_2_C_4_)_4_ at 5, 25, and 37 °C at pH 4.7. The vertical scale of the spectral region indicated by the dotted box has been enlarged three-fold. (**c**) Imino region of ^1^H NMR spectra of d(G_2_C_4_)_8_ at 5 °C and pH 4.7, 6.0, and 7.2. (**d**) Imino region of ^1^H NMR spectra of d(G_2_C_4_)_8_ at 5, 25, and 37 °C at pH 4.7. The vertical scale of the spectral region indicated by the dotted box has been enlarged three-fold.

^1^H NMR spectra of d(G_2_C_4_)_4_ and d(G_2_C_4_)_8_ at a constant temperature of 5 °C and pH ranging from 4.7 to 7.2 showed signals between δ 16.1 and 16.8 ppm corresponding to hemi-protonated C^+^-C base pairs as well as signals indicative of Watson-Crick hydrogen bonding (Fig. 2a,c). With increasing pH, imino signals from hemi-protonated C^+^-C base pairs decreased while signals corresponding to GC-base pairs remained constant. Furthermore, at pH 4.7, d(G_2_C_4_)_8_ exhibited a broad signal at δ 15.3 ppm, which is indicative of i-motif structure formation (Fig. 2c)^18^. In the case of d(G_2_C_4_)_4_ at pH 4.7, a broad signal at δ 15.5 ppm was first observed at 25 °C (Fig. 2b). For both oligonucleotides signals corresponding to i-motifs further increased in intensity up to 37 °C while signals between δ 16.1 and 16.8 ppm gradually decreased at higher temperatures and eventually vanished at 37 °C (Fig. 2b,d). Signals indicative of GC-base pairs persisted under all tested temperatures.

**Figure 3:**
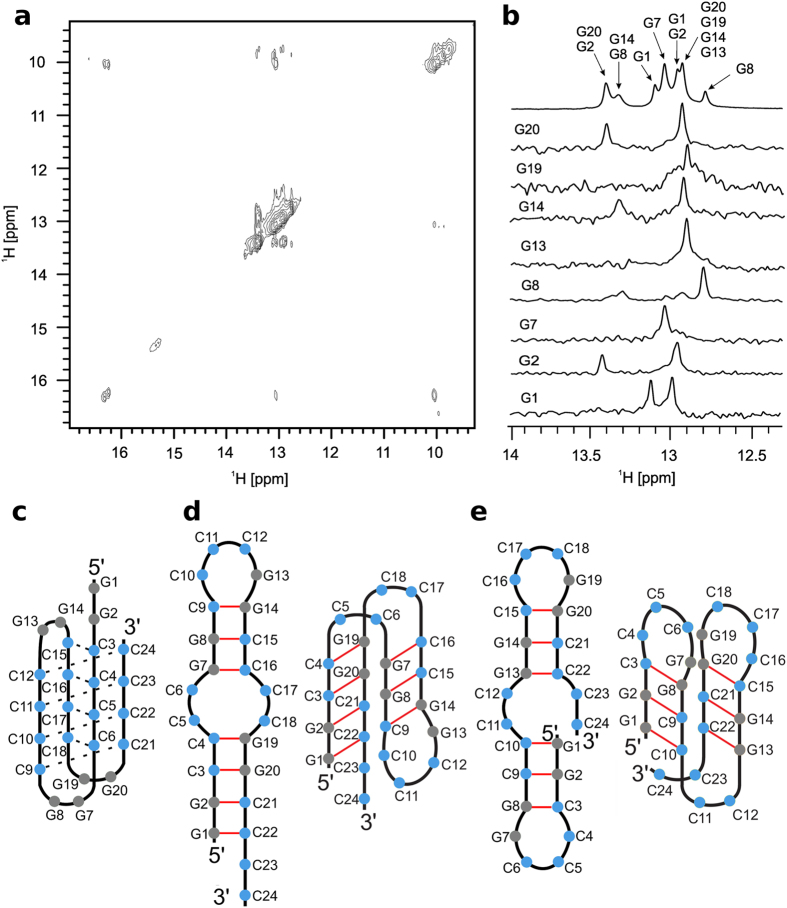
Assignment of guanine imino protons and proposed structures. (**a**) Imino-imino region of NOESY spectrum of d(G_2_C_4_)_4_ at mixing time of 150 ms. (**b**) Assignment of imino protons of guanine residues involved in GC base pairs using 1D ^15^N-edited HSQC NMR spectra of residue specific (marked on the left side of each spectrum) ^15^N, ^13^C-isotopically labelled d(G_2_C_4_)_4_. Top spectrum in B represents imino region of ^1^H NMR spectrum. All spectra were acquired at pH 4.7 and 5 °C. (**c**) Proposed structures of i-motif and (**d**,**e**) hairpins adopted by d(G_2_C_4_)_4_.

Intra or intermolecular nature of structures adopted by d(G_2_C_4_)_4_ and d(G_2_C_4_)_8_ was established by measuring translation diffusion coefficients (D_t_) in 90% H_2_O/10% ^2^H_2_O at 25 °C. The observed D_t_ values range between 1.3–1.4 and 0.8–0.9 × 10^−6^ cm^2^ s^−1^ for d(G_2_C_4_)_4_ and d(G_2_C_4_)_8_, respectively. The corresponding hydrodynamic dimensions are consistent with a unimolecular nature of structures adopted by both d(G_2_C_4_)_4_ and d(G_2_C_4_)_8_ in solution^28^,^29^. Analysis of 2D NOESY NMR spectrum of d(G_2_C_4_)_4_ acquired at pH 4.7 and 5 °C (Fig. 3a) showed that signals corresponding to hemi-protonated C^+^-C base pairs exhibit NOE correlations with signals of GC-base pairs. Therefore, C^+^-C and GC-base pairs are in proximity within a single structure. On the contrary, no NOE correlations between signals at δ 15.5 ppm and signals of GC-base pairs were observed, which suggested presence of another distinct structure. Observed NOE correlations together with translation diffusion coefficient value of d(G_2_C_4_)_4_ indicate the coexistence of i-motifs with guanine residues in loops (Fig. 3c) and protonated hairpins involving GC and C^+^-C base pairs. 1D ^15^N-edited HSQC NMR spectra on partially ^15^N-residue-specific labelled d(G_2_C_4_)_4_ were used to determine which guanine residues were involved in GC-base pairs within protonated hairpins^30^. NMR data clearly showed that all guanine residues were within GC-base pairs since signals of their imino protons appear in the region indicative of Watson-Crick hydrogen bonds (Fig. 3b). It is interesting to note that single imino signals were observed for guanine residues G7, G13 and G19 in 1D ^15^N-edited HSQC NMR spectra, while two imino signals per guanine residue were observed for the others (Fig. 3b). The presence of two imino signals for some of guanine residues could be explained only by the coexistence of two different protonated hairpins, with a difference in the involvement of guanine residues of their GC-base pairs and their loop region (Fig. 3d,e).

**Figure 4:**
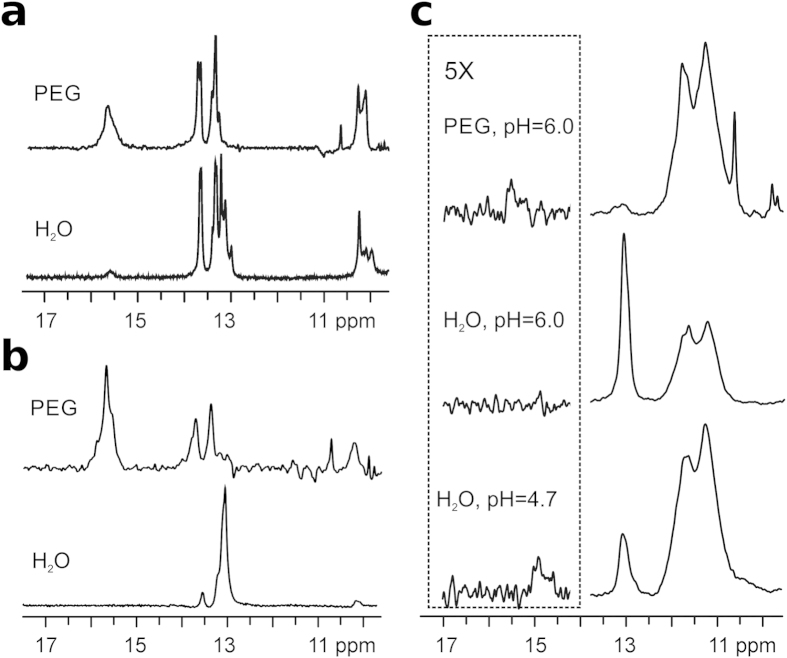
i-motifs persist under molecular crowding and in the presence of the complementary strand. (**a**) Imino region of ^1^H NMR spectra of d(G_2_C_4_)_4_ and (**b**) d(G_2_C_4_)_8_ in water and in 40% w/v PEG8000, at pH 6.0 with 100 mM K^+^ ions. (**c**) Imino region of ^1^H NMR spectra of equimolar mixture of d(G_2_C_4_)_8_ and d(G_4_C_2_)_8_ in water at pH 4.7 and 6.0, and in 40% w/v PEG8000 at pH 6.0 with 100 mM K^+^ ions. The vertical scale of the spectral region indicated by the dotted box has been enlarged five-fold. All spectra were recorded at 37 °C.

In addition to molecular crowding, other factors including biologically important cations can affect non-B DNA structures (e.g. K^+^ ion presence promotes formation of G-quadruplexes). In order to simulate intracellular conditions, d(G_2_C_4_)_4_ and d(G_2_C_4_)_8_ were dissolved in 40% w/v PEG8000 with added 100 mM K^+^ ions and 37 °C. Under these conditions and at pH approaching neutral values, ^1^H NMR spectra of d(G_2_C_4_)_4_ and d(G_2_C_4_)_8_ exhibit strong broad signals corresponding to i-motifs in addition to signals indicating the presence of hairpins connected through GC-base pairs (Fig. 4a,b). Intensities of signals belonging to hemi-protonated C^+^-C base pairs within i-motifs were significantly higher under molecular crowding conditions in comparison to the same signals observed for both oligonucleotides dissolved in water alone.

Since *in vivo* both G- as well as C-rich strands are present, an equimolar mixture of d(G_4_C_2_)_8_ and d(G_2_C_4_)_8_ was dissolved in water in the presence of 100 mM K^+^ ions at pH 4.7 and 37 °C. Imino region of the resulting mixture after annealing exhibited signals characteristic of Hoogsteen hydrogen bonding within G-quartets in addition to signals corresponding to GC-base pairs. A weak signal at δ 15.0 ppm, indicating the presence of i-motifs, was also observed at pH 4.7 (Fig. 4c, bottom). Upon the increase of pH to 6.0 this signal disappeared and only signals corresponding to imino protons involved in G-quartets and GC-base pairs were observed (Fig. 4c, middle). However, a weak signal at δ 15.5 ppm indicating i-motifs was observed at pH 6.0 and 37 °C under molecular crowding conditions in the presence of K^+^ ions and the sense d(G_4_C_2_)_8_ strand (Fig. 4c, top). Therefore, an equimolar mixture of the complementary strands in conditions simulating the intracellular environment forms G-quadruplexes, hairpins and i-motifs, rather than a DNA duplex.

## Discussion

Here we have shown that the highly C-rich anti-sense strand of HREM can form protonated hairpins as well as i-motifs and that the formation of these structures takes place at physiological pH, temperature and under molecular crowding conditions. In addition we show that these structures persist even in the presence of the complementary strand, which has important implications for key cellular processes – DNA replication and transcription. The potential separation of the HREM complementary strands independently of the replication/transcription machinery may adversely affect both the stability and rate of the HREM replication and transcription contributing to the proposed disease mechanisms.

Currently, attempts are underway to influence the disease mechanisms through targeting of G-quadruplex structures and their binding proteins on the sense-strand (G_4_C_2_)_n_ HREM^31^,^32^. Since the transcript of the *C9ORF72* mRNA is made on the basis of the anti-sense strand, the latter may also be targeted for regulation of the transcription leading to RNA toxicity and DPR accumulation. So far, several ligands that either stabilize or destabilize specific i-motif/hairpin structures have been developed^33^. Proof of concept for transcriptional modulation through targeting of i-motif/hairpins was recently demonstrated in the case of *BCL2* gene, whose i-motif/hairpin conformation pair was first characterized as part of its promotor region in 2009^22^,^34^. HREM i-motifs/hairpins could therefore also prove as attractive new target for modulation of all three proposed disease mechanisms in *C9ORF72*-associated ALS and FTLD at their source.

## Methods

### Sample preparation

Reverse-phase purified oligonucleotides d(G_2_C_4_), d(G_2_C_4_)_2_, d(G_2_C_4_)_4,_ d(G_4_C_2_)_4_, and dual HPLC purified oligonucleotides d(G_2_C_4_)_8_ and d(G_4_C_2_)_8_ were purchased from Eurogentec (Seraing, Belgium). Oligonucleotides d(G_2_C_4_) and d(G_2_C_4_)_2_ were cleaned using 2M LiCl and dialysed four times against water, and concentrated using an ultra-filtration device (Merck Millipore, Herfordshire, UK) and an ultra-filtration membrane (regenerated cellulose, Millipore). Oligonucleotides d(G_2_C_4_)_4,_ d(G_4_C_2_)_4_, d(G_2_C_4_)_8_, and d(G_4_C_2_)_8_ were cleaned using 2M LiCl and passed through an Amicon ultrafilter. Residue specific 10% ^15^N, ^13^C-guanine and cytosine labeled samples were synthesized on K&A Laborgeraete GbR DNA/RNA Synthesizer H-8 using standard phosphoramidite chemistry. Deprotection was performed with overnight incubation in 20% aqueous ammonia at 50 °C. 2M LiCl was added prior to purification and concentration of samples using an Amicon ultrafilter.

The samples were lyophilized overnight and diluted in 90% of H_2_O and 10% of ^2^H_2_O. The concentrations of the 300 μL NMR samples were 3.3, 2.7, 1.1, 0.7, 0.4 and 0.3 mM for d(G_2_C_4_), d(G_2_C_4_)_2_, d(G_2_C_4_)_4,_ d(G_4_C_2_)_4_, d(G_2_C_4_)_8_, and d(G_4_C_2_)_8_ respectively. In case of equimolar mixing experiments, equimolar quantities of d(G_2_C_4_)_4_ with d(G_4_C_2_)_4_ and d(G_2_C_4_)_8_ with d(G_4_C_2_)_8_ were heated to 90 °C prior to annealing. For testing of additional conditions d(G_2_C_4_)_4,_ d(G_4_C_2_)_4_, d(G_2_C_4_)_8_, and d(G_4_C_2_)_8_ were diluted to 0.1 mM. For CD measurements, samples d(G_2_C_4_), d(G_2_C_4_)_2_ and d(G_2_C_4_)_4_ were diluted 60-fold, while d(G_2_C_4_)_8_ was diluted 30-fold in respective diluents (see CD section). For molecular crowding experiments, samples were prepared by diluting to 0.1 mM in 40% w/v PEG (8000 MW) (Sigma Aldrich, Munich, Germany) in water. 10% residue specific labeled samples were diluted in 90% of H_2_O and 10% of ^2^H_2_O. Concentrations were ranging from 0.4 to 0.7 mM. The pH of samples was adjusted by the addition of LiOH or HCl and measured using the 780 pH Meter (Metrohm, Herisau, Switzerland).

### Circular dichroism spectroscopy

CD spectra were recorded on an Applied Photophysics Chirascan CD spectrometer at 5, 25, or 37 °C using a 0.1 cm path length quartz cell. The wavelength was varied from 200 to 320 nm. Three scans were averaged for each CD spectrum. In each case corresponding blanks were used for baseline correction. For CD spectroscopy the initial samples were measured 20 days after dilution at 5, 25, and 37 °C, at pH 6.2, 6.2, 6.5 and 6.5 for d(G_2_C_4_), d(G_2_C_4_)_2_, d(G_2_C_4_)_4_, and d(G_2_C_4_)_8_, respectively. The initial CD measurement was performed after denaturation at 90 °C. Measurements at different pH were performed in H_2_O at 37 °C and pH 4.0, 4.7, 5.0, 6.0, 6.5, 7.0, 7.2, 8.0, and 8.5. In order to model molecular crowding conditions, samples were prepared by diluting d(G_2_C_4_)_4_ and d(G_2_C_4_)_8_ to 15 μM per strand in 40% w/v PEG (8000 MW). The pH of the PEG solutions in water was adjusted using LiOH or HCl. All samples with molecular crowding conditions were measured at 37 °C after 8 days.

### UV spectroscopy

Melting experiments of d(G_2_C_4_), d(G_2_C_4_)_2_, d(G_2_C_4_)_4_, and d(G_2_C_4_)_8_ were performed on a Varian Cary 100 Bio UV-VIS spectrometer (Varian Inc.) equipped with a thermoelectric temperature controller. UV melting experiments were performed on samples diluted to 2 μM in 100mM K-phosphate buffer at pH 6.0, using 1 cm path-length quartz cells. A combination of mineral oil and a fixed cuvette cap was used to prevent evaporation and sample loss due to high temperatures. A stream of nitrogen was applied throughout the measurements to prevent condensation at lower temperatures. Folding/unfolding processes were followed between 10 and 90 °C by measuring absorbance at 260 nm using scanning rates of 0.5 and 0.1 °C min^−1^. Temperatures of half transition (T_1/2_) were determined using the first derivative method.

### Native PAGE electrophoresis

Native gel electrophoresis of d(G_2_C_4_), d(G_2_C_4_)_2_, d(G_2_C_4_)_4,_ d(G_4_C_2_)_4_, d(G_2_C_4_)_8_, d(G_4_C_2_)_8_ and equimolar mixtures of d(G_2_C_4_)_4_ with d(G_4_C_2_)_4_, and d(G_2_C_4_)_8_ with d(G_4_C_2_)_8_, respectively, was performed on a 15% polyacrylamide gel (5 °C at 100 V) in 1xTBE (pH 6.5) buffer with 100 mM KCl. 1nmol of DNA sample was mixed with loading buffer (3 μl 15% ficoll, 2.5 X Tris borate) and diluted to 20 μl with water. The approximate size of the bands was determined by using the GeneRuler Ultra Low range DNA Ladder (Thermo Scientific, Waltham, MA USA). The samples were treated at room temperature overnight before loading, except for the equimolar mixture samples, that were heated to 90 °C and either cooled immediately on ice or cooled slowly at room temperature prior to loading. Following the overnight electrophoresis, the gel was stained with Stains All gel stain solution (Sigma Aldrich) and filmed using the DNR Bio-Imaging Systems instrument.

### NMR spectroscopy

All NMR spectra were obtained with Agilent Technologies DD2 600 MHz NMR spectrometer at 5, 25, and 37 °C using a triple resonance cold probe. Standard 1D ^1^H spectra were acquired with the use of DPFGSE, watergate 3919 or PRESAT solvent suppression. Diffusion coefficient measurements were performed by a spin-echo pulse sequence with PFG gradient strengths between 0.49 and 29.06 G cm^−1^. NOESY spectra were acquired with mixing times of 80 and 150 ms. Assignment of imino protons of guanine residues was done by 1D ^15^N-edited HSQC experiments performed on residue specific 10% ^15^N, ^13^C-isotopically labeled oligonucleotides. Measurements at different pH were performed at pH 4.7, 6.0 and 7.2. Measurements under molecular crowding conditions were performed using 40% w/v dPEG (8000 MW) (Polymer Source Inc., Dorval, Canada) in water 7 days after annealing. KH_2_PO_4_ was used in experiments with 100 mM K^+^ ions. NMR spectra were processed and analyzed using VNMRJ (Varian Inc.) and Sparky (UCSF) software.

## Additional Information

**How to cite this article**: Kovanda, A. *et al*. Anti-sense DNA d(GGCCCC)_n_ expansions in C9ORF72 form i-motifs and protonated hairpins. *Sci. Rep.* **5**, 17944; doi: 10.1038/srep17944 (2015).

## Supplementary Material

Supplementary Information

## References

[b1] van der Zee J. . A Pan-European Study of the *C9orf72* Repeat Associated with FTLD: Geographic Prevalence, Genomic Instability, and Intermediate Repeats. Hum. Mutat. 34, 363–373 (2013). 23111906 10.1002/humu.22244 PMC3638346

[b2] DeJesus-Hernandez M. . Expanded GGGGCC hexanucleotide repeat in noncoding region of C9ORF72 causes chromosome 9p-linked FTD and ALS. Neuron 72, 245–256 (2011). 21944778 10.1016/j.neuron.2011.09.011 PMC3202986

[b3] Renton A. E. . A hexanucleotide repeat expansion in C9ORF72 is the cause of chromosome 9p21-linked ALS-FTD. Neuron 72, 257–268 (2011). 21944779 10.1016/j.neuron.2011.09.010 PMC3200438

[b4] Beck J. . Large C9orf72 Hexanucleotide Repeat Expansions Are Seen in Multiple Neurodegenerative Syndromes and Are More Frequent Than Expected in the UK Population. Am. J. Hum. Genet. 92, 345–353 (2013). 23434116 10.1016/j.ajhg.2013.01.011 PMC3591848

[b5] Gómez-Tortosa E. . C9ORF72 hexanucleotide expansions of 20–22 repeats are associated with frontotemporal deterioration. Neurology 80, 366–370 (2013). 23284068 10.1212/WNL.0b013e31827f08ea

[b6] Smith B. N. . The C9ORF72 expansion mutation is a common cause of ALS+/–FTD in Europe and has a single founder. Eur. J. Hum. Genet. EJHG 21, 102–108 (2013). 22692064 10.1038/ejhg.2012.98 PMC3522204

[b7] van Blitterswijk M. . Association between repeat sizes and clinical and pathological characteristics in carriers of C9ORF72 repeat expansions (Xpansize-72): a cross-sectional cohort study. Lancet Neurol. 12, 978–988 (2013). 24011653 10.1016/S1474-4422(13)70210-2 PMC3879782

[b8] Vatovec S., Kovanda A. & Rogelj B. Unconventional features of C9ORF72 expanded repeat in amyotrophic lateral sclerosis and frontotemporal lobar degeneration. Neurobiol. Aging 35, 2421.e1–2421.e12 (2014). 10.1016/j.neurobiolaging.2014.04.015 24836899

[b9] Haeusler A. R. . C9orf72 nucleotide repeat structures initiate molecular cascades of disease. Nature 507, 195–200 (2014). 24598541 10.1038/nature13124 PMC4046618

[b10] Pettersson O. J., Aagaard L., Jensen T. G. & Damgaard C. K. Molecular mechanisms in DM1 - a focus on foci. Nucleic Acids Res. 43, 2433–2441 (2015). 25605794 10.1093/nar/gkv029 PMC4344492

[b11] Mori K. . The C9orf72 GGGGCC repeat is translated into aggregating dipeptide-repeat proteins in FTLD/ALS. Science 339, 1335–1338 (2013). 23393093 10.1126/science.1232927

[b12] Mizielinska S. . C9orf72 repeat expansions cause neurodegeneration in Drosophila through arginine-rich proteins. Science 345, 1192–1194 (2014). 25103406 10.1126/science.1256800 PMC4944841

[b13] Kwon I. . Poly-dipeptides encoded by the C9orf72 repeats bind nucleoli, impede RNA biogenesis, and kill cells. Science 345, 1139–1145 (2014). 25081482 10.1126/science.1254917 PMC4459787

[b14] Fratta P. . C9orf72 hexanucleotide repeat associated with amyotrophic lateral sclerosis and frontotemporal dementia forms RNA G-quadruplexes. Sci. Rep . 2, 1016 (2012). 23264878 10.1038/srep01016 PMC3527825

[b15] Reddy K., Zamiri B., Stanley S. Y. R., Macgregor R. B. & Pearson C. E. The disease-associated r(GGGGCC)n repeat from the C9orf72 gene forms tract length-dependent uni- and multimolecular RNA G-quadruplex structures. J. Biol. Chem. 288, 9860–9866 (2013). 23423380 10.1074/jbc.C113.452532 PMC3617286

[b16] Šket P. . Characterization of DNA G-quadruplex species forming from C9ORF72 G4C2-expanded repeats associated with amyotrophic lateral sclerosis and frontotemporal lobar degeneration. Neurobiol. Aging 36, 1091–1096 (2015). 25442110 10.1016/j.neurobiolaging.2014.09.012

[b17] Gehring K., Leroy J. L. & Guéron M. A tetrameric DNA structure with protonated cytosine.cytosine base pairs. Nature 363, 561–565 (1993). 8389423 10.1038/363561a0

[b18] Benabou S., Aviñó A., Eritja R., González C. & Gargallo R. Fundamental aspects of the nucleic acid i-motif structures. RSC Adv . 4, 26956 (2014).

[b19] Qin Y. & Hurley L. H. Structures, folding patterns, and functions of intramolecular DNA G-quadruplexes found in eukaryotic promoter regions. Biochimie 90, 1149–1171 (2008). 18355457 10.1016/j.biochi.2008.02.020 PMC2585383

[b20] Kendrick S. & Hurley L. H. The role of G-quadruplex/i-motif secondary structures as cis-acting regulatory elements. Pure Appl. Chem. Chim. Pure Appl . 82, 1609–1621 (2010). 10.1351/PAC-CON-09-09-29 PMC3142959 21796223

[b21] Brooks T. A., Kendrick S. & Hurley L. Making sense of G-quadruplex and i-motif functions in oncogene promoters. FEBS J. 277, 3459–3469 (2010). 20670278 10.1111/j.1742-4658.2010.07759.x PMC2971675

[b22] Kendrick S. . The dynamic character of the BCL2 promoter i-motif provides a mechanism for modulation of gene expression by compounds that bind selectively to the alternative DNA hairpin structure. J. Am. Chem. Soc. 136, 4161–4171 (2014). 24559410 10.1021/ja410934b PMC3985915

[b23] Kypr J., Kejnovská I., Renciuk D. & Vorlícková M. Circular dichroism and conformational polymorphism of DNA. Nucleic Acids Res. 37, 1713–1725 (2009). 19190094 10.1093/nar/gkp026 PMC2665218

[b24] Vorlíčková M., Kejnovská I., Bednářová K., Renčiuk D. & Kypr J. Circular dichroism spectroscopy of DNA: from duplexes to quadruplexes. Chirality 24, 691–698 (2012). 22696273 10.1002/chir.22064

[b25] Miyoshi D., Karimata H. & Sugimoto N. Hydration regulates thermodynamics of G-quadruplex formation under molecular crowding conditions. J. Am. Chem. Soc. 128, 7957–7963 (2006). 16771510 10.1021/ja061267m

[b26] Cui J., Waltman P., Le V. H. & Lewis E. A. The effect of molecular crowding on the stability of human c-MYC promoter sequence I-motif at neutral pH. Mol. Basel Switz . 18, 12751–12767 (2013). 10.3390/molecules181012751 PMC6270392 24132198

[b27] Dai J., Ambrus A., Hurley L. H. & Yang D. A Direct and Nondestructive Approach To Determine the Folding Structure of the I-Motif DNA Secondary Structure by NMR. J. Am. Chem. Soc. 131, 6102–6104 (2009). 19400591 10.1021/ja900967r PMC2749488

[b28] Marusic M., Sket P., Bauer L., Viglasky V. & Plavec J. Solution-state structure of an intramolecular G-quadruplex with propeller, diagonal and edgewise loops. Nucleic Acids Res. 40, 6946–6956 (2012). 22532609 10.1093/nar/gks329 PMC3413137

[b29] Trajkovski M., da Silva M. W. & Plavec J. Unique structural features of interconverting monomeric and dimeric G-quadruplexes adopted by a sequence from the intron of the N-myc gene. J. Am. Chem. Soc. 134, 4132–4141 (2012). 22303871 10.1021/ja208483v

[b30] Phan A. T. & Patel D. J. A Site-Specific Low-Enrichment ^15^ N, ^13^ C Isotope-Labeling Approach to Unambiguous NMR Spectral Assignments in Nucleic Acids. J. Am. Chem. Soc. 124, 1160–1161 (2002). 11841271 10.1021/ja011977m

[b31] Zamiri B., Reddy K., Macgregor R. B. Jr & Pearson C. E. TMPyP4 porphyrin distorts RNA G-quadruplex structures of the disease-associated r(GGGGCC)n repeat of the C9orf72 gene and blocks interaction of RNA-binding proteins. J. Biol. Chem. 289, 4653–4659 (2014). 24371143 10.1074/jbc.C113.502336 PMC3931028

[b32] Lagier-Tourenne C. . Targeted degradation of sense and antisense C9orf72 RNA foci as therapy for ALS and frontotemporal degeneration. Proc. Natl. Acad. Sci. U. S. A. 110, E4530–4539 (2013). 24170860 10.1073/pnas.1318835110 PMC3839752

[b33] Day H. A., Pavlou P. & Waller Z. A. E. i-Motif DNA: Structure, stability and targeting with ligands. Bioorg. Med. Chem. 22, 4407–4418 (2014). 24957878 10.1016/j.bmc.2014.05.047

[b34] Kendrick S., Akiyama Y., Hecht S. M. & Hurley L. H. The i-motif in the bcl-2 P1 promoter forms an unexpectedly stable structure with a unique 8:5:7 loop folding pattern. J. Am. Chem. Soc. 131, 17667–17676 (2009). 19908860 10.1021/ja9076292 PMC2787777

