# Supplementary material for: Anti-sense DNA d(GGCCCC)n expansions in C9ORF72 form i-motifs and protonated hairpins
Source: Sci Rep. 2015 Dec 3;5:17944. doi: 10.1038/srep17944 (PMC4668579; doi:10.1038/srep17944)
Supplement: Supplementary Information [file srep17944-s1.pdf]

Supplementary Information

Anti-sense DNA d(GGCCCC)$_n$ expansions in C9ORF72 form i-motifs and protonated hairpins

Anja Kovanda [1,‡] , Matja Zalar [2,‡], Primož Šket [2,3], Janez Plavec [2,3,4]* & Boris Rogelj [1,5]*

[1] Department of Biotechnology, Jožef Stefan Institute, Jamova 39, SI-1000 Ljubljana, Slovenia.
[2] Slovenian NMR Centre, National Institute of Chemistry, Hajdrihova 19, SI-1000 Ljubljana, Slovenia.
[3] EN-FIST Center of Excellence, Trg OF 13, SI-1000 Ljubljana, Slovenia.
[4] Faculty of Chemistry and Chemical Technology, University of Ljubljana, Večna pot 113, SI-1000 Ljubljana, Slovenia.
[5] Biomedical Research Institute BRIS, Puhova 10, SI-1000 Ljubljana, Slovenia.
* Corresponding authors: Janez Plavec (janez.plavec@ki.si) or Boris Rogelj (boris.rogelj@ijs.si)
[‡] These authors contributed equally to this work.

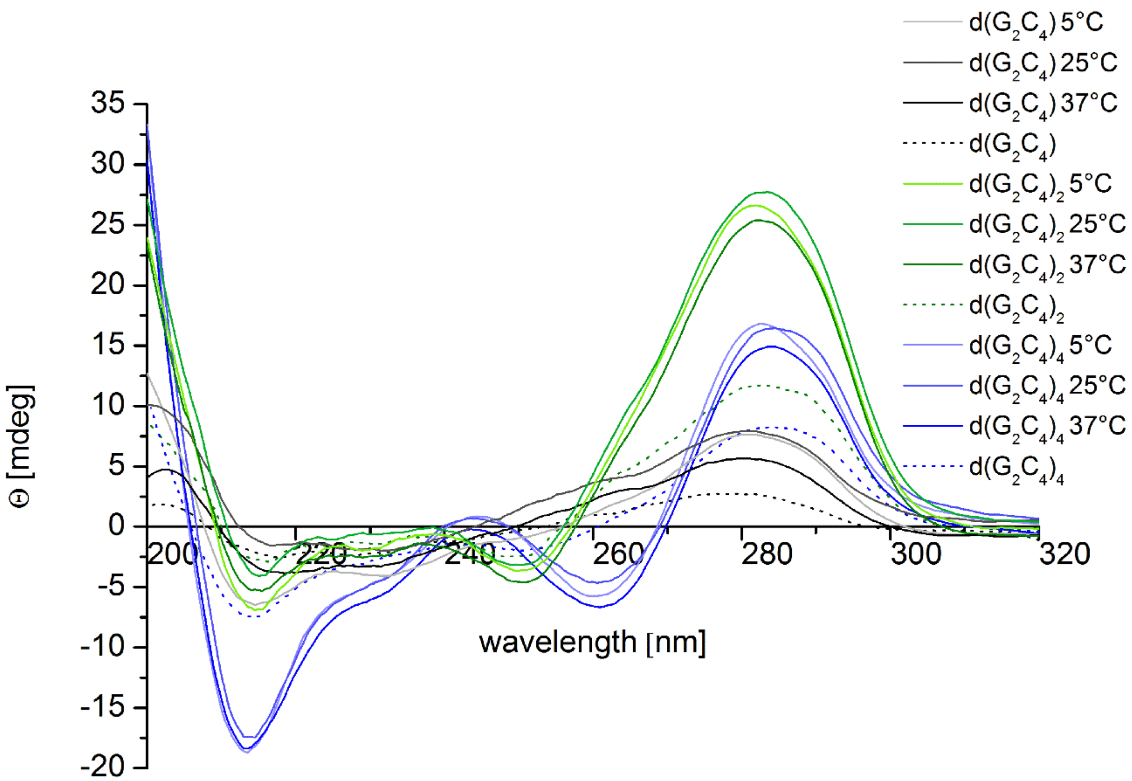

Supplementary Fig. 1. CD spectra of d(G_2C_4) (black), d(G_2C_4)$_2$ (green), and d(G_2C_4)$_4$ (blue) immediately after dilution in H_2O (dotted lines) and after incubation (full lines) at 5, 25, and 37 °C.

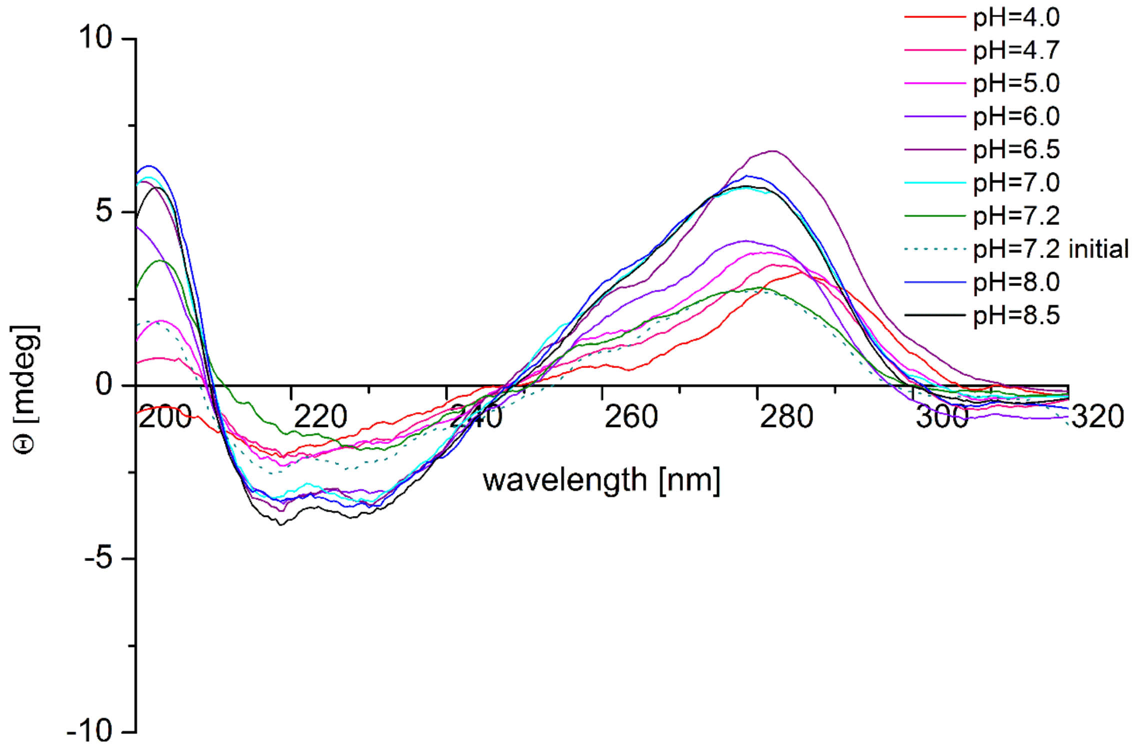

Supplementary Fig. 2. CD spectra of d(G_2C_4) at different pH values in H_2O at 37 °C.

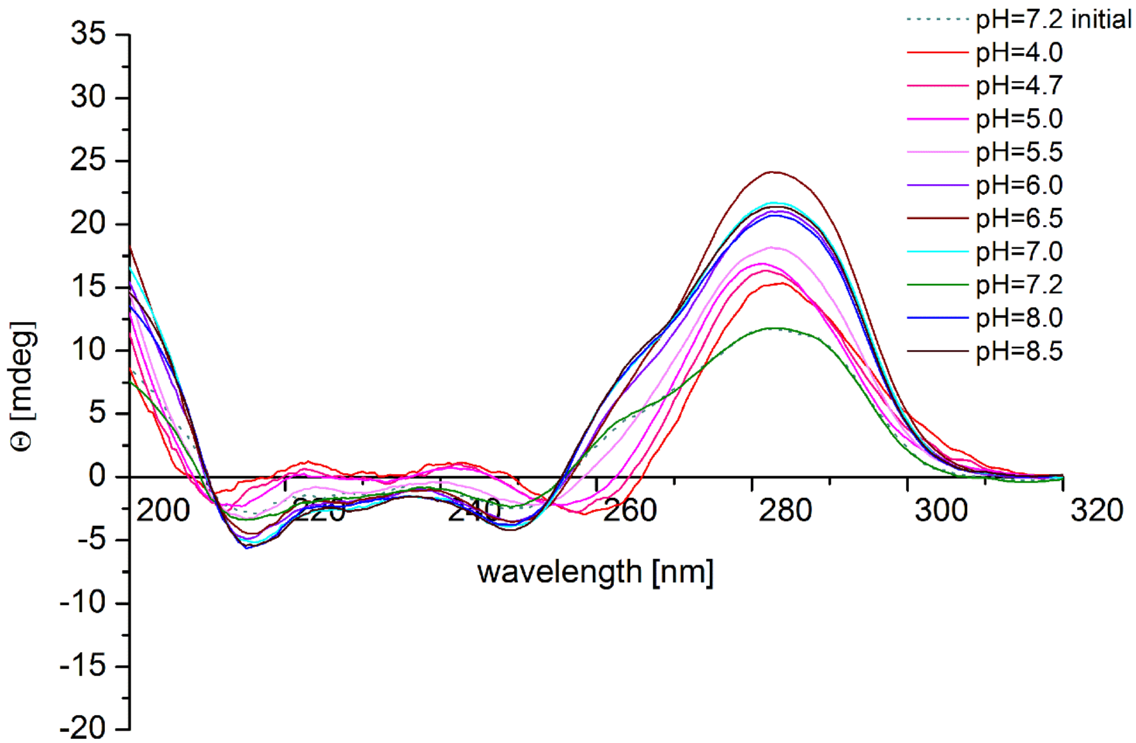

Supplementary Fig. 3. CD spectra of d(G_2C_4)$_2$ at different pH values in H_2O at 37 °C.

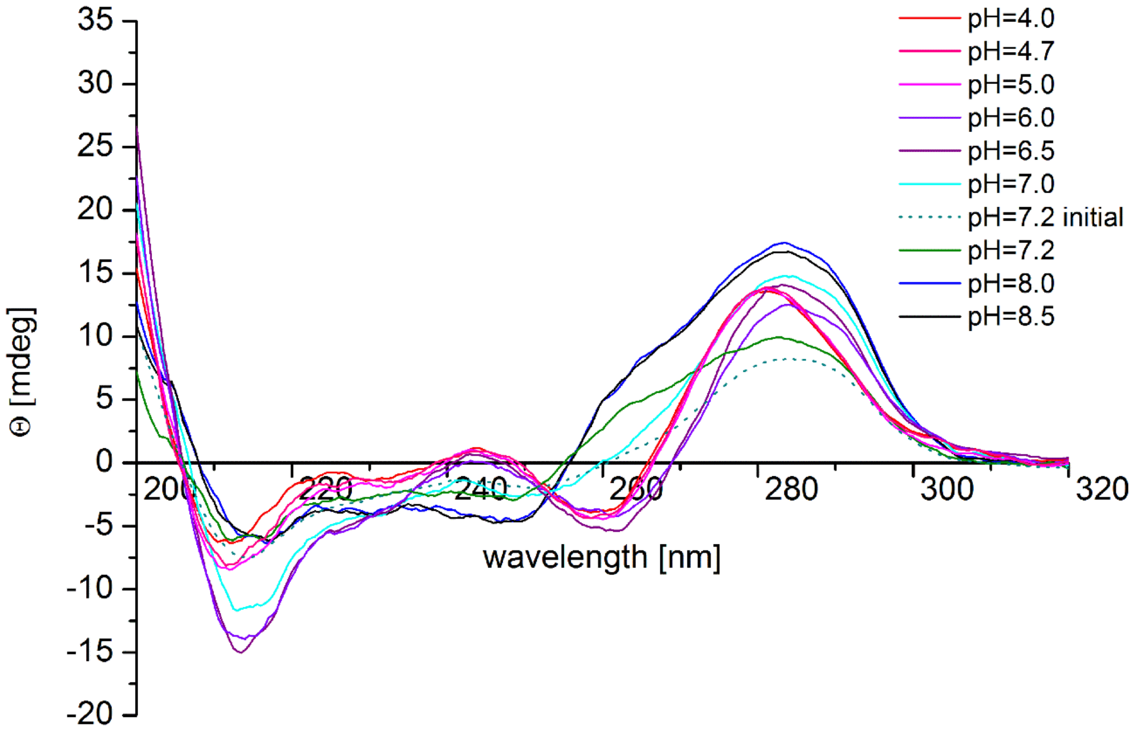

Supplementary Fig. 4. CD spectra of d(G_2C_4)$_4$ at different pH values in H_2O at 37 °C.

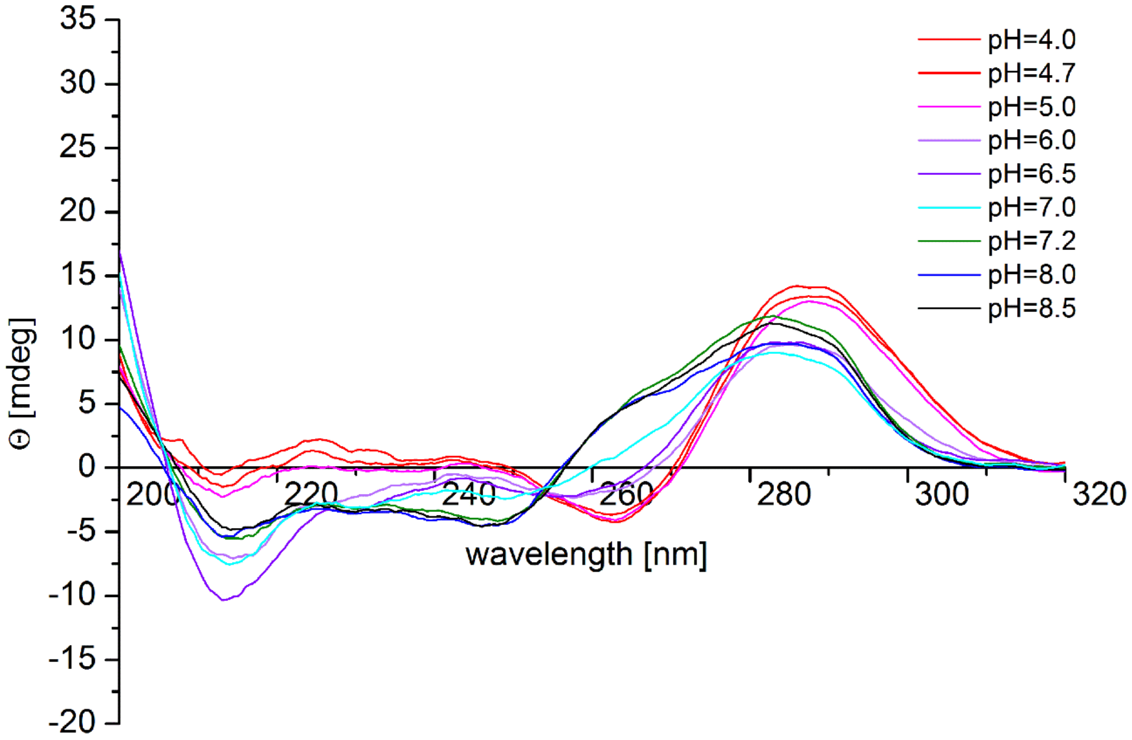

Supplementary Fig. 5. CD spectra of $d(G_2C_4)_8$ at different pH values in H_2O at 37 °C

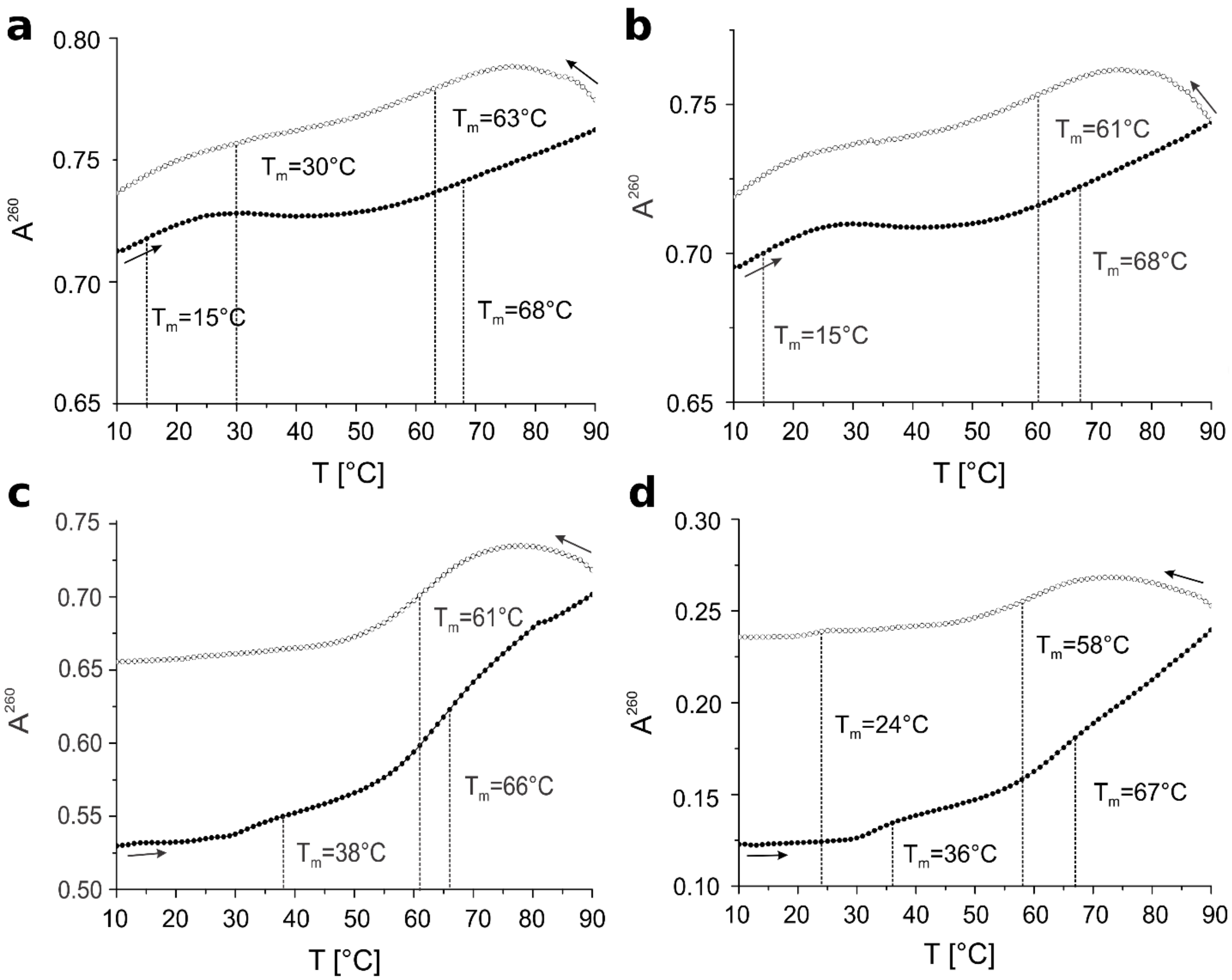

Supplementary Fig. 6. a, UV melting curves of d(G_2C_4), **b,** d(G_2C_4)$_2$, **c,** d(G_2C_4)$_4$, **d,** and d(G_2C_4)$_8$ in 100 mM K^+-phosphate buffer at pH 6.0. For oligonucleotides d(G_2C_4) and d(G_2C_4)$_8$, two transitions have been observed upon both heating and cooling, while d(G_2C_4)$_2$ and d(G_2C_4)$_4$ exhibited a single transition upon cooling in contrast to two transitions occurring upon heating. In all cases, upon heating the first transition occurred below 40 °C, which was followed by the second one at around 67 °C. It is noteworthy that processes of unfolding and refolding may proceed through different intermediates. Importantly, $T_{1/2}$ values are reproducible upon repetition of heating and cooling cycles. Observed multiple transitions indicate presence of different structures in solution.

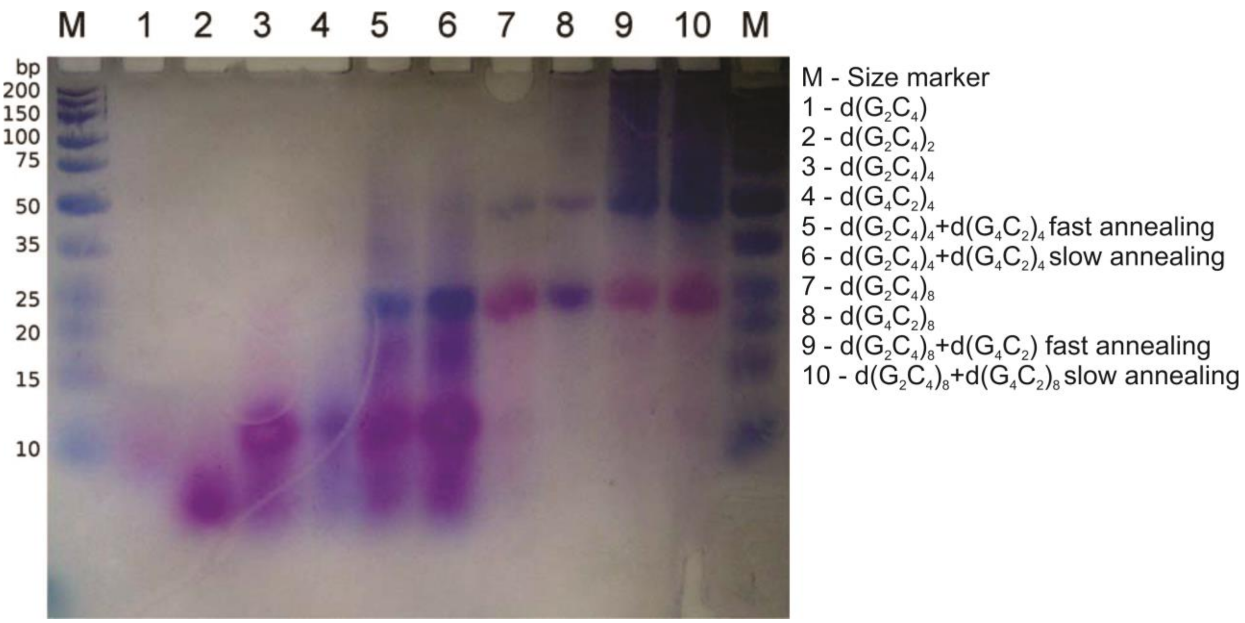

Supplementary Fig. 7. Native PAGE electrophoresis of $d(G_2C_4)$ (lane 1), $d(G_2C_4)_2$ (lane 2), $d(G_2C_4)_4$ (lane 3), $d(G_4C_2)_4$ (lane 4), equimolar mixture of $d(G_2C_4)_4$ and $d(G_4C_2)_4$ cooled immediately (lane 5) or slowly (lane 6), $d(G_2C_4)_8$ (lane 7), $d(G_4C_2)_8$ (lane 8), equimolar mixture of $d(G_2C_4)_8$ and $d(G_4C_2)_8$ cooled immediately (lane 9) or cooled slowly (lane 10) at pH 6.5. Thermo Scientific GeneRuler Ultra Low range DNA Ladder was used as a size marker (M). The $d(G_2C_4)$ traveled to just under the 10-bp marker band (lane 1), suggesting that the single repeat unit forms inter-molecular structures. The $d(G_2C_4)_2$ (lane 2) traveled further suggesting it forms an intra-molecular hairpin species. $d(G_2C_4)_4$ and the sense $d(G_4C_2)_4$ travelled in a similar manner between the 10- and 15-bp marker bands with some smearing visible below 10-bp (lanes 3, 4). The main species of $d(G_2C_4)_4$ traveling at approx. 12-bp exhibited similar mobility as the G-quadruplex formed by $d(G_4C_2)_4$. The additional smearing below 10-bp marker band furthermore suggests that $d(G_2C_4)_4$ also forms a folded hairpin structure. The strand mixing experiments with fast and slow cooling show that in the presence of $d(G_2C_4)_4$ and $d(G_4C_2)_4$ complementary strands, classic double helix 24-bp DNA strand is formed (lanes 5, 6) in addition to intra-molecular structures of both strands, with visible species down to below the 10-bp marker band. Both $d(G_2C_4)_8$ and $d(G_4C_2)_8$ showed bands at 48-bp as well as between the 20- and 25-bp marker bands (lanes 7, 8). The latter, faster mobility species of $d(G_2C_4)_8$ and $d(G_4C_2)_8$ probably consist of i-motifs and G-quadruplexes, respectively while the slower mobility species could represent homo-duplexes. In the case of $d(G_2C_4)_8$ and $d(G_4C_2)_8$ mixing experiment, the expected 48-bp duplex is visible in addition to species between the 20- and 25-bp marker bands, associated with the presence of intra-molecular single-stranded structures (lanes 9, 10).

Supplementary Table 1. Temperatures of half-transition in UV melting experiments

	$T_{1/2}$ (heating) [°C]		$T_{1/2}$ (cooling) [°C]	
**$d(G_2C_4)$**	15	68	63	30
**$d(G_2C_4)_2$**	15	68	61	
**$d(G_2C_4)_4$**	38	66	61	
**$d(G_2C_4)_8$**	36	67	58	24
